# Increasing the Utility of the Comprehensive Assessment of Psychopathic Personality–Lexical Rating Scale (CAPP-LRS): Instrument Adaptation and Simplification

**DOI:** 10.1177/10731911211040108

**Published:** 2021-08-20

**Authors:** Katherine B. Hanniball, Richard Hohn, Erin K. Fuller, Kevin S. Douglas

**Affiliations:** 1 Simon Fraser University, Burnaby, British Columbia, Canada

**Keywords:** psychopathy, Comprehensive Assessment of Psychopathic Personality, Lexical Rating Scale, instrument adaptation, instrument accessibility

## Abstract

The Comprehensive Assessment of Psychopathic Personality–Lexical Rating Scale (CAPP-LRS) is a self-report instrument designed to index psychopathy according to the CAPP psychopathy framework. Developed with the expressed goal of advancing the state of knowledge regarding the specific features of psychopathy, the CAPP model and associated instruments have garnered increasing attention and support in the field. Despite the conceptual strength of the CAPP model, the advanced lexical structure of its primary research tool (the CAPP-LRS) has led researchers to question the utility of the instrument for use with some populations of interest (e.g., forensic/correctional and adolescent/young adult samples). The aim of the present work was to address this issue by creating a lexically simplified, though functionally equivalent, version of the CAPP-LRS to increase accessibility to critically relevant populations. A set of two studies (*N* = 602) describes the adaptation protocol and the initial validation of the modified instrument.

Psychopathic personality disorder, or psychopathy, is an important forensic construct that has received extensive scholarly and empirical attention due to its relevance within legal and clinical settings. Despite being one of the more well-known and researched personality disorders, psychopathy is a concept shrouded in controversy. Prominent debates within the field range from questions concerning the relationship between criminal behavior and psychopathy (Cooke & Michie, 2001; Hare & Neumann, 2010; Skeem & Cooke, 2010), to the relevance, or lack thereof, of the putatively adaptive aspect of Boldness/Fearless Dominance (Lilienfeld et al., 2012; Miller & Lynam, 2012), to fundamental disagreements concerning the structure of the disorder and its presentation across populations (e.g., Cooke et al., 2007; Cooke & Michie, 2001; Edens et al., 2006; Harris et al., 1994; Haslam et al., 2012; Lilienfeld et al., 2012; Miller & Lynam, 2012; Olver et al., 2013).

Disagreement surrounding the proper conceptualization of psychopathy and the utility of its assessment has led to fierce debate and discord in the operationalization and measurement of the disorder; thus, to date, numerous instruments have been created to index and assess its presence across multiple modalities. Interview-based protocols such as the Psychopathy Checklist–Revised (PCL-R; Hare, 1991, 2003) and its progeny (PCL-SV; Hart et al., 1995; PCL-YV; Forth et al., 2003) rank among the more well-known, validated, and widely used instruments in the literature. However, the advent of research demonstrating sound validity and reliability of self-report psychopathy measures (especially for research purposes) has established the value of tools that rely on subjective self-ratings and reports to assess the presence of psychopathic personality (Ray et al., 2013).

## The Comprehensive Assessment of Psychopathic Personality (CAPP)

Scholars and researchers in the areas of psychology and philosophy of science have long recognized the necessity of theoretical explication of constructs prior to their operationalization and measurement (Blashfield & Livesley; 1991; Cooke et al., 2012; Westen & Rosenthal, 2005). In line with this approach, Cooke et al. (2012) developed the CAPP, a framework designed to reconcile some of the aforementioned controversies in the field and increase conceptual clarity regarding the defining features of psychopathy. In the first stage of development, the authors engaged in a systematic review of existing empirical and theoretical literature and conducted interviews with subject matter experts in order to develop an overinclusive item list of facets belonging to the essential definition of psychopathy (Cooke et al., 2012; Guttman & Greenbaum, 1998). The list was refined and narrowed to include only those symptoms that were specific to personality pathology as opposed to behavioral outcomes (e.g., symptoms reflecting deviance at the individual rather than cultural or social level). Items were also chosen to reflect the dynamic nature of pathology expression, thereby allowing sensitivity to any change that might occur over time and fluctuation from a measurement perspective.

On selection, primary symptoms were translated into natural (nontechnical) language, with meaning triangulated by a series of three synonymous adjectives. The resulting model contained 33 trait descriptive primary symptoms (e.g., “Garrulous,” “Domineering”) rationally organized into six thematic domains of personality functioning including: *Attachment*, *Behavioral*, *Cognitive*, *Dominance*, *Emotional*, and *Self* (for descriptions, see Cooke et al., 2012). As noted by the CAPP authors, this model represents an “ideal type” or prototypical model of psychopathy, designed to help understand and evaluate different operationalizations of psychopathic personality disorder, and serve as a foundation for new insights, conceptualizations, and operations (Cooke et al., 2012). Primary advantages of the model include its comprehensibility, simplicity (i.e., items defined in natural language and constitutive of basic-level traits), and efficiency. The use of single adjectives or adjectival phrases also reduces subjective interpretation on behalf of the administrator or examinee in its avoidance of complex, wordy, or morality-laden descriptors.

To date, the CAPP model has sustained operationalization across multiple assessment modalities (e.g., structured interview and third-party rating scale: Cooke et al., 2004; self-report: Sellbom et al., 2019) including the CAPP-Lexical Rating Scale (CAPP-LRS; Cooke et al., 2012), an instrument designed primarily for conceptual/construct driven research. As a research tool, the CAPP-LRS exhibits strict fidelity to the original CAPP-model and is operationalized as a brief self-rating symptom checklist directly mapping the original 33 primary CAPP symptoms (e.g., “Aggressive,” “Inflexible”). The CAPP-LRS has now been successfully translated into more than 25 languages (Cooke, 2018), suggesting support for the conceptual strength of the model and attesting to the stability of its content across diverse cultures (Saucier & Goldberg, 2001). Furthermore, prototypicality research with the CAPP-LRS has allowed evaluation of the 33 primary CAPP symptoms with respect to their relevance and centrality for the psychopathy construct, with results suggesting that forensic mental health experts and community members alike rate the majority of CAPP symptoms as highly prototypical of psychopathy (Hoff et al., 2012; Kreis et al., 2012).^1^ These findings have been found to hold relatively consistently across gender, age, and several language groups (Flórez et al., 2018; Hoff et al., 2014; Kreis & Cooke, 2011; Sörman et al., 2014), suggesting that the CAPP model may be less biased than other existing conceptualizations of psychopathic personality. In addition to conceptual and content-based evaluations, studies examining the reliability, adequacy, and psychometric properties of CAPP instruments have suggested these measures exhibit moderate to high levels of internal consistency across several relevant populations and have demonstrated sound convergent validity with other established measures of psychopathic personality (Dawson et al., 2012; Hanniball et al., 2018, 2019; Pedersen et al., 2010; Sandvik et al., 2012; Sellbom et al., 2015). From a structural perspective, most research with the CAPP model has favored a three-factor solution over the originally proposed six-factor model (Hanniball et al., 2020; Sellbom et al., 2015); findings which hold constant when tested in diverse cultures (Flórez et al., 2018). Such research indicates that a model comprising the three essential elements of interpersonal dominance, behavioral impulsivity, and deficient affective experience adequately captures psychopathy as measured under the CAPP framework.

Despite strong evidence for the theoretical foundation of the CAPP model, a recurrent criticism of the primary research instrument associated with the CAPP conceptual framework (the CAPP-LRS) is the advanced reading level and linguistic sophistication required to complete the self-rating scale. For example, although the model uses natural language, accurate ratings on items such as “Garrulous” or “Self-Aggrandizing” are unlikely to be achievable for individuals possessing lower verbal comprehension skills or lacking in advanced formal education. This feature of the CAPP-LRS hampers its utility for research among arguably some of the most relevant and understudied populations in examining the CAPP model. For instance, the lack of explicit reference to criminal and antisocial behavior in the CAPP-LRS makes it a highly useful tool for examining psychopathic personality among antisocial populations, maintaining a focus on trait pathology as opposed to behavioral outcomes inherent to the population under study (e.g., violence, criminality). Furthermore, the primary symptoms contained in the CAPP model (and reflected in the CAPP-LRS) were selected, in part, for their dynamic nature, allowing for sensitivity to change and fluctuation. This characteristic positions the CAPP-LRS well for research examining treatment/intervention efficacy, but also for extending study into younger populations (e.g., adolescents/young adults) to examine the expression of psychopathic trait pathology across the lifespan.^2^

## Test Adaptations and Linguistic Accommodations

An essential feature in the development of any valid assessment instrument—whether for research, clinical, or applied purposes—includes attempts to reduce construct irrelevant variance, or the introduction of extraneous variables that affect the meaningfulness of assessment outcomes (Messick, 1995). Situated within the framework of test validity theory, certain features of an instrument (e.g., elevated linguistic demand) can introduce construct irrelevant variance when uniformly applied across populations who possess the required capacity and those who do not. For example, though the CAPP-LRS in its current form may provide a useful mechanism for assessing psychopathic personality traits within samples of university students or within corporate settings, its utility for accurately assessing the construct under study becomes more questionable when applied to populations possessing lower levels of education, verbal skills, and decreased cognitive capacity (e.g., some forensic populations; adolescents). Given the relevance of these populations for the study of psychopathic personality, the systematic modification and simplification of the CAPP-LRS research tool would fill a gap in the literature and provide a highly useful contribution for continued research of psychopathy, allowing for the removal of construct irrelevant barriers to assessment, while maintaining integrity to the construct under study.

Examining literature drawn from the area of standardized academic achievement testing among English learners provides some insight into best practices for the modification of assessment instruments to reduce construct irrelevant linguistic complexity. For example, a large body of research has examined the impact of minor wording changes in test items to clarify the construct under assessment (e.g., scientific/mathematical knowledge) in academic settings among students with lower verbal skills, with results demonstrating that syntactic and vocabulary complexity has a significant impact on performance (Abedi et al., 2005; Abedi & Lord, 2001; Noble, et al., 2020; Rivera & Stansfield, 2001). An important feature of successful linguistic accommodation concerns maintaining the integrity and validity of the assessment; that is, such modifications should not compromise the construct under study. In practical terms, this means that simplifications directed toward reducing the English language load of test items should not differentially affect the performance of those individuals possessing sufficient verbal skills to complete the assessment without modification (Koenig & Bachman, 2004; Sireci et al., 2003).

Fortunately, several studies examining such modifications in high stakes testing arenas (e.g., standardized academic achievement/educational placement evaluations) suggest that though performance gaps narrow when high-quality linguistic modifications are applied, simplification of test items does not affect the performance of those who have a stronger grasp of the English language (Abedi et al., 2005; Abedi & Lord, 2001; Noble et al., 2020; see also Abedi et al., 2004, for a general discussion). Evaluation of the modification procedures included in such studies reveal several features of successful linguistic simplification, and specific suggestions for language alteration while maintaining fidelity to content include: using familiar or more frequently used words, present tense, and concrete rather than abstract formats (Abedi et al., 1997). Research also suggests that using words that are short (simple morphologically), and omitting or replacing words containing nuanced meaning, technical jargon, and colloquialisms results in lowered linguistic demand for examinees (Abedi et al., 2005).

## Current Study

The present study sought to address gaps in the literature by modifying the existing CAPP-LRS to simplify the linguistic structure of the tool. In its current format, the primary research instrument associated with the CAPP framework exhibits limited utility for use within populations that do not possess highly advanced verbal skills (McCuish et al., 2019; Sellbom et al., 2019). Indeed, results of the Flesch-Kincaid Grade Level Test suggest that the grade level required for reasonable comprehension of the items included in the CAPP-LRS is 20.4 years of education, suggesting that those individuals who have not graduated college are unlikely to fully understand the measure.^3^ While there are advantages to understanding the distribution of psychopathic traits among more normative samples (e.g., university students, community members), arguably the most relevant populations for the study of psychopathy are composed of persons within forensic/psychiatric settings, where the ratios of individuals possessing elevated levels of psychopathic features are higher than in the general population (Hare & Neumann, 2008). As individuals within these settings generally possess lower levels of formal education, decreased access to opportunities, and decreased verbal and cognitive skills (Greenberg et al., 2007; Keyes et al., 2017), self-ratings on an instrument such as the CAPP-LRS may be inaccurate and error-laden, thereby introducing construct irrelevant linguistic complexity and diluting clarity in measurement.

Further populations, as of yet relatively understudied from the perspective of the CAPP model, are those of youth, adolescents, and young adults. Given the proposed dynamic nature of the trait symptomology included in the CAPP model, and the reduced reliance on behavioral indicators as found in other instruments, this framework is well situated to examine the nature of psychopathic personality from a developmental perspective, providing insight into the expression of psychopathic personality across the lifespan. However, given the more limited cognitive and verbal abilities of adolescents as compared to adults, the current linguistic structure of the CAPP-LRS may pose obstacles for the utility of the tool to study self-ratings among such populations. As such, the present study sought to address implementation barriers to the use of the CAPP-LRS for continued research of psychopathic personality by conducting a systemic modification of the tool to reduce linguistic complexity while maintaining the integrity of the construct under study.

Recent research has demonstrated the utility of self-report assessments of psychopathic personality, suggesting that such ratings exhibit adequate to strong convergence with informant reports (e.g. ~ *r* = .30 to .70; Fowler & Lilienfeld, 2007; Lynam & Widiger, 2007; Miller et al., 2011), and provide a valid and reliable means of assessing trait distribution within populations of interest, while offering an economical alternative to the more time and resource intensive investigations associated with interview and third party rater protocols (Ray et al., 2013). Recently, Sellbom et al. (2019) developed a self-report instrument corresponding to the CAPP framework (CAPP-SR). Though still in its infancy, initial validation work is promising, suggesting evidence of convergence with other established measures of psychopathy, and correspondence with results of prototypically studies conducted on the CAPP model (Sellbom et al., 2019). Consequently, the development of the CAPP-SR represents a highly promising advance in CAPP research. Despite its clear contribution to the literature, the recently developed CAPP-SR comprises 99 items making it a more time intensive enterprise than the CAPP-LRS symptom checklist and demanding increased effort on the behalf of examinees.

Given that willingness to complete a time intensive questionnaire may depend on certain personality traits commonly associated with psychopathy (e.g., problems with concentration; conscientiousness), increased demand may result in inadvertent selection bias in the sample under study. Furthermore, the translation of the symptom checklist into self-report statements that, by necessity, contain moral undertones introduces additional social desirability concerns not included in the CAPP-LRS, a feature that can pose problems when assessing personality pathology in disorders such as psychopathy (Lilienfeld & Fowler, 2006). Finally, a lexically simplified or “sheltered” version of the CAPP-LRS has the advantage of maintaining strict fidelity to the structure and design of the original CAPP research tool, providing a direct downward translation of the existing CAPP-LRS, and making it an economic choice for continued research on the CAPP model in situations where time and resources are limited.

## Adapting the CAPP-LRS

The process of scale adaptation and lexical simplification was guided in part by consultation with linguistic experts at Simon Fraser University (see next for full description). Two samples of undergraduate university students were recruited for the purposes of item selection and scale development (Pilot Study), and scale evaluation and validation (Validation Study). The use of these samples allows us to establish that the simplified instrument performs as well as the original and more complex CAPP-LRS among those with higher language skills. This first step allows for comparison with previous work with the CAPP-LRS and sets the stage for further validation studies within other samples. The protocol for this study was approved by the research ethics board at Simon Fraser University, and all participants provided informed consent. We report below how we determined our sample size, data exclusions, all manipulations, and all measures in the study.

## Method: Pilot Study

### Participants and Procedures

In the first step of the modification protocol, a synonym list for each of the 33 primary CAPP-LRS items was derived. To develop a synonym selection strategy, several researchers and professors of linguistics at a large university in Canada were contacted for opinion. Guidance indicated the use of dictionary synonym generation tools and latent sematic index word-mapping to generate replacement candidates for each symptom. Subsequent to generation of a synonym list, adult native language speakers were recommended as adjudicators to rank synonym similarity. Each CAPP-LRS item, alongside 5 top-ranked synonyms (selected by cross-referencing items deemed by the authors to most closely approximate the target word) was included in an anonymous survey posted through the Research Participation System at Simon Fraser University. All participants received one course credit in return for their participation. In order to obtain a measure of perceived difficulty, participants were asked to rank the readability/difficulty of synonym and original item, as well as the similarity between the original item and each synonym. Items were rated on a scale of 1 (easiest) to 5 (most difficult) for difficulty, as well as similarity (1 “least similar” to 5 “most similar”). A total of 49 undergraduate participants who rated English as their first language competed the survey.

## Pilot Study Analyses and Results

As a primary step, mean difficulty ratings of original CAPP-LRS items were evaluated by the authors. Replacement of original CAPP-LRS items was deemed necessary if the mean of the item fell above the mid-point on a five-point scale (i.e., greater than 3). Second, one-sample *t* tests were employed to compare the means of synonyms of lesser ranked difficulty with original items and only replacement synonyms with significantly lower difficulty ratings than original items were retained. All retained item synonyms were then considered in terms of their similarity to the original CAPP item and the item with the highest similarity ranking was selected as the replacement item. Of the 33 original CAPP items, 23 items required revision subsequent to the initial difficulty screen (see Table S1, available in the online supplement). The two “easiest” CAPP-LRS synonym replacements retained subsequent to one-sample *t* test analyses were then evaluated for their similarity to the original item in the second step of item selection. The final item list was derived by selecting the single synonym replacement item with the lowest difficulty rating and the highest similarity score (see Table 1). We again utilized the Flesch-Kincaid Grade Level Test to provide an estimate of the number of years of education required for comprehension of the revised items. Results indicated that these items required roughly 8.6 years of education for reasonable comprehension, suggesting that the CAPP-Basic required nearly 12 years fewer formal education for comprehension that the original CAPP-LRS (which, again, was 20.4). Such findings indicate that the measure is likely usable with adolescents in Grades 8 and above, and will be more accessible for use with forensic populations within which Grade 8 is the highest level of education for many individuals (Correctional Service of Canada, 1995, 1996).

**Table 1.** Final Item Replacement List.

Original CAPP-LRS item	Domain	Replacement item
Detached	Attachment	Cold
Uncommitted	Attachment	Independent
Unempathetic	Attachment	Unfeeling
Uncaring	Attachment	Unkind
Lacks Perseverance	Behavioral	Gives up easily
Reckless	Behavioral	Irresponsible
Aggressive	Behavioral	Nasty
Unreliable	Behavioral	—
Restless	Behavioral	—
Disruptive	Behavioral	—
Lacks Concentration	Cognitive	Lacks focus
Intolerant	Cognitive	Unfair
Inflexible	Cognitive	Stubborn
Lacks Planfulness	Cognitive	Leaves things to chance
Suspicious	Cognitive	—
Antagonistic	Dominance	Unfriendly
Domineering	Dominance	Bossy
Deceitful	Dominance	False
Manipulative	Dominance	Tricky
Insincere	Dominance	Fake
Garrulous	Dominance	Long-winded
Lacks Anxiety	Emotional	Unworried
Lacks Remorse	Emotional	Shameless
Lacks Pleasure	Emotional	—
Lacks Emotional Depth	Emotional	—
Lacks Emotional Stability	Emotional	—
Self-Aggrandizing	Self	Snooty
Sense of Entitlement	Self	Sense of being owed things
Sense of Invulnerability	Self	Sense of being unbeatable
Self-Justifying	Self	Offers excuses for behavior
Unstable Self-Concept	Self	Unsteady sense of self
Self-Centered	Self	—
Sense of Uniqueness	Self	—

*Note.* Blank spaces indicate that the original item was retained. CAPP-LRS = Comprehensive Assessment of Psychopathic Personality–Lexical Rating Scale.

## Method: Validation Study

### Procedure

Once the final items had been selected for the CAPP-LRS revision (i.e., the CAPP-Basic), the second step of the modification protocol sought to evaluate the revised scale in terms of its functional similarity to the instrument, its psychometric properties, and criterion validity. To do so, a sample of university undergraduate students was recruited through the Research Participation System at the same university as that in the pilot study. Participant eligibility was restricted to students who had not completed the pilot study and were fluent in the English language. All participants completed basic demographic information and were administered the CAPP-LRS and CAPP-Basic (counterbalanced presentation). Participants also completed several scales assessing theoretically relevant external criteria, including self-report measures of aggression, sensation seeking, and impulsivity. All participants were remunerated with a single course credit. On completion of the survey, participants were debriefed. Interested readers may contact the first author for research materials.

It should be noted that the potential for biased responding and positive impression management is elevated in this study, as the characteristics being measured are generally considered socially aversive (i.e., traits associated with psychopathy). However, this concern is mitigated somewhat due to the complete anonymity afforded by the online sampling method, thereby reducing the likelihood that response bias would significantly affect the validity of self-reports (Watts et al., 2016). Furthermore, in the present study, participants were unaware of the construct under measurement (e.g., psychopathy) as survey instructions suggested content was related to personal experiences. Finally, the structure of the original and revised CAPP-LRS scales alleviate some concerns regarding socially desirable responding, as the items are adjectival symptoms (e.g. “Uncommitted”) as opposed to value-laden or threatening questions (e.g. “It doesn’t bother me to see someone else in pain;” for discussion, see Sudman & Bradburn, 1974).

### Sample

A total of 553 undergraduate university students were recruited for participation in the validation study (*M_age_* = 19.31; range: 17-41). A sample size of at least 500 was determined in advance of data collection to allow sufficient power to complete our primary analyses (Jiang et al., 2016; Reise & Yu, 1990). Three quarters of the sample self-identified as female (75%), and the largest portion of the sample indicated Caucasian descent (28.4%), followed by those endorsing Chinese (24.7%), and South Asian (17.7%) ethnic identities. Also represented to a lesser degree were individuals who self-identified as African American, Filipino, Japanese, Korean, Southeast Asian, and West Asian. All participants rated English as their first or second language. Interested readers may contact the first author for research materials.

### Measures

#### CAPP-LRS

As noted, the CAPP-LRS (Cooke et al., 2012) is a self-rating instrument designed to index the CAPP conceptual model of psychopathy. The self-rating instrument contains 33 trait descriptive adjectival symptoms identified as relevant to psychopathy, which are grouped rationally into six thematic domains of personality functioning including *Attachment*, *Behavioral*, *Cognitive*, *Dominance*, *Emotional*, and *Self.* As reviewed above, although originally proposed to cover the noted six distinctly thematic content areas, the majority of previous empirical work on the CAPP model has demonstrated that a three factor model comprising the domains of *Dominance*, *Disinhibition*, and *Deficient Emotional/Interpersonal Attachment* frequently offers a better interpretation of the data across multiple samples, and fits more comfortably with longstanding theory regarding the conceptual contours of psychopathy (Cooke & Michie, 2001; Flórez et al., 2018; Hanniball et al., 2020; Lim, 2016; Sellbom et al., 2015). As such, both models were considered here.

Within the present sample, reliability and item homogeneity for the original six-factor model was excellent for the total score, and generally ranged from the lower end of adequate to good (Clark & Watson, 2016; Nunnally & Bernstein, 1994) at the domain level. One exception emerged, with the *Emotional* domain evidencing lower reliability than witnessed in past work (Total: α = .92, measure of internal consistency [MIC] = .26; *Attachment*: α = .71, MIC = .29; *Behavioral*: α = .68; MIC = .27; *Cognitive*: α = .65, MIC = .25; *Dominance*: αα = .81, MIC = .42; *Emotional*: α = .53, MIC = .19; *Self*: α = .69, MIC = .26). Consistent with previous research, and as expected given scale length, the three-factor model fared better when reliability was estimated with coefficient alpha, and was similar when evaluated according to MIC values, with domain level reliability ranging from the upper end of acceptable to good (*Disinhibition*: α = .76, MIC = .27; *Deficient Attachment*: α = .83, MIC = .33; *Dominance*: α = .85, MIC = .32).

#### CAPP-Basic

The revised version of the CAPP-LRS mirrors the format of the original version, though the trait descriptive symptoms are linguistically simplified to make the instrument accessible to a wider population. Though the 33 primary symptoms in the revised scale have been modified to reach individuals with lower levels of reading capacity, the three additional synonyms presented alongside the primary items in the original version remain the same to ensure consistency and coherence with the original instrument. As such, the six domains reflected in the original scale remain the same. In general, reliability and item homogeneity estimates were similar for the total score. Further, though certain domains evidenced substantially lower coefficient alpha values under the revised version, with the exception of the Attachment domain, MIC values remained fairly consistent. This finding is important because indices such as coefficient alpha are heavily reliant on the number of indicators in a test, thereby limiting its utility in establishing the unidimensionality of a scale. The average interitem correlation on the other hand does not rely on the number of items in a scale (see Briggs & Cheek, 1986; Cortina, 1993; Clark & Watson, 2016), thereby making it a much more straightforward measure of internal consistency (Total: α = .91, MIC = .24; *Attachment*: α = .50, MIC = .21; *Behavioral*: α = .69, MIC = .30; *Cognitive*: αα = .59, MIC = .23; *Dominance*: α = .72, MIC = .32; *Emotional*: α = .53, MIC = .19; *Self*: α = .67, MIC = .24). As with the original scale, modeling the CAPP under a three-factor structure proved superior, with scale reliabilities falling in the acceptable to good range (*Disinhibition*: α = .76, MIC = .26; *Deficient Attachment*: α = .79, MIC = .28; *Dominance*: α = .81, MIC = .27).

#### The Brief Sensation Seeking Scale (BSSS)

The BSSS (Hoyle et al., 2002) is a brief, self-report measure of sensation seeking tendencies adapted from the longer Sensation Seeking Scale (Zuckerman et al., 1978). The brief version of the scale contains eight items which are combined into a total score representing overall sensation seeking tendencies. Within the present sample adequate reliability and item homogeneity was observed (Total: α = .81 MIC = .35).

#### The Buss-Perry Aggression Questionnaire–Short Form (BPAQ-SF)

The BPAQ-SF (Diamond et al., 2005) is a brief 12-item scale derived from the longer Buss-Perry Aggression Questionnaire, which has exhibited sound validity and reliability in past work, and possesses the same factor structure as the original form (Diamond & Magaletta, 2006), assessing *Physical Aggression*, *Anger*, *Hostility*, and *Verbal Aggression*, as well as General Aggression in the form of a total score. Within the present sample, acceptable reliability and item homogeneity was observed for all domains (α’s = .69 to .79, MIC’s = .36 to .56) as well as for the total score (α = .87, MIC = .36).

#### The Barratt Impulsiveness Scale–Brief (BIS-Brief)

The BIS-Brief (Steinberg et al., 2013) is an eight-item version of the 30-item original Barratt Impulsiveness Scale parent version (Patton et al., 1995). The Brief version of the parent scale provides a unidimensional measure of impulsivity that has exhibited strong psychometric properties in past work. In the present sample, the instrument displayed good reliability and item homogeneity (α = .72, MIC = .25).

### Data Analytic Plan

To examine the functional comparability of the CAPP-LRS and CAPP-Basic we employed a multifaceted analytic strategy, the first step of which aimed to establish the comparability of the two instruments’ internal psychometric properties. A second step aimed to compare the external validity of the two instruments. A final third step aimed to compare the instruments descriptively and inferentially to one another.

With regard to comparing the instruments psychometrically, we employed both confirmatory factor analytic (CFA) and item response theory (IRT) techniques to assess the overall factor structure of the two instruments, as well as understand the specific characteristics (e.g., difficulty, discrimination) of each item. We chose to employ this approach because each set of techniques offers unique advantages depending on the level (e.g., test, domain, item) of the instrument under psychometric scrutiny. Using CFA, we were able to evaluate the overall factor structure of the CAPP-LRS and CAPP-Basic to confirm whether our sample adequately fit with the three- and six-factor CAPP models specified in previous research (e.g., Hanniball et al., 2020; Lim, 2016),^4^ allowing us to ascertain whether the alterations made for the CAPP-Basic led to any degradation of those models’ fit compared with the CAPP-LRS. CFA also allowed us to compare the item loadings for each model to determine whether the lexical alterations of the CAPP-Basic potentially resulted in a different pattern of item relationships with their respective factors. Previous CAPP research has also heavily favored CFA techniques for test-level evaluations (see Flórez et al., 2018; Hanniball et al., 2020; Sellbom et al., 2015), such that incorporating them into our analysis plan allowed for the most direct comparison with past research. In conducting the CFAs, we employed weighted least squares means- and variance-adjusted estimation, given that the CAPP-LRS and CAPP-Basic items were ordinal and nonnormally distributed. The factors were allowed to correlate for both the three- and six-factor models. Model fit indices were considered adequate when comparative fit index (CFI) ≥ .90, Tucker–Lewis index (TLI) ≥ .90, root mean square error of approximation (RMSEA) < .08, and standardized root mean residual (SRMR) < .08, given the number of items loading on each factor (see Browne & Cudeck, 1992; Hu & Bentler, 1999).

We further favored including an IRT approach to evaluating the instruments at the domain (i.e., factor) and item levels. Specifically, we plotted the test information curves (TICs) and conditional standard error of measurement (CSEM) estimates of domain-specific unidimensional graded-response models to determine how well each set of domain items functioned across the latent continuums of their respective domains. At the item level, IRT parameter estimates, in particular estimates of item discrimination, were evaluated and compared between the two instruments. Item characteristic curves (ICCs) and item information curves (IICs) were also plotted and visually assessed to compare the characteristics of item responding of each instrument, as well as measurement precision of each item across the latent continuum of their respective domains. Taken together, the wealth of psychometric information provided by this multifaceted approach facilitated a much more comprehensive and nuanced comparison between the CAPP-LRS and CAPP-Basic than either set of techniques could afford in isolation.

With regard to assessing each instruments’ external validity, we evaluated and compared the criterion validity of both scales by examining the association and predictive capabilities of both instruments with relevant outcome criteria. To do so, we conducted bivariate zero-order correlations between each domain and participants’ self-report scores of aggression, sensation seeking, and impulsivity. We followed up these analyses with a series of Ordinary Least Squares (OLS) regressions, controlling for the effects of gender and ethnicity within the models.^5^ Finally, in regard to comparing the scores of the CAPP-LRS and CAPP-Basic, we calculated various descriptive statistics of their total and subscale (i.e., domain) scores. Additionally, we conducted paired-sample *t* tests to evaluate whether average total scores of the CAPP-LRS and CAPP-Basic significantly differed within our sample. Paired-samples *t* test we also conducted to compare the average scores across each subscale.

## Results: Validation Study

### Internal Psychometric Properties

#### Confirmatory Factor Analyses

CFAs of the six-factor first-order model mapping directly on the theorized structure of the CAPP, and examination of the polychoric correlation matrices for both instruments suggested high levels of redundancy and poor fit, rendering the model inadmissible. CFAs for the three-factor first-order models confirmed in previous work (Hanniball et al., 2020; Lim, 2016), were then fit to the data. Although the CAPP-Basic, χ^2^ = 1228.530, degrees of freedom (*df*) = 492, *p <* .001, CFI = .908, TLI = .901, SRMR = .085, RMSEA = .066, RMSEA 90% confidence interval (CI) [.061, .070]) was found to have slightly worse model fit compared with the CAPP-LRS (χ^2^ = 1082.619, *df* = 492, *p <* .001, CFI = .926, TLI =.920, SRMR = .082, RMSEA = .059, RMSEA 90% CI [.054, .063]), both models demonstrated adequate fit across most indices, with only the estimates of the SRMRs exceeding their suggested cut-offs. The three factors were highly and similarly correlated among each other for both the CAPP-LRS and CAPP-Basic models (Table 2).

**Table 2.** CAPP-LRS Factor Correlations.

Factor	1	2	3
CAPP-LRS			
1. Dominance	—	[0.827, 0.911]^a^	[0.816, 0.914]^a^
2. Disinhibition	.869***	—	[0.829, 0.922]^a^
3. Deficient Attachment	.865***	.875***	—
CAPP-LRS			
1. Dominance	—	[0.811, 0.910]^a^	[0.898, 0.964]^a^
2. Disinhibition	.861***	—	[0.833, 0.930]^a^
3. Deficient Attachment	.931***	.881***	—

*Note*. CAPP-LRS = Comprehensive Assessment of Psychopathic Personality–Lexical Rating Scale.

a Values reflect a 95% confidence interval.

***p < .001. (Fisher, 1992).

Item-level estimates, including standardized item loading and *R*^2^ estimates for both the CAPP-LRS and CAPP-Basic models, as well as their differences, can be found in Table 3. Overall, a near-identical pattern of loading significance and similar pattern of loading magnitude was observed between the CAPP-LRS and CAPP-Basic models, with the notable exception of one item; when changed from “Uncommitted” in the CAPP-LRS to “Independent” in the CAPP-Basic, the item evidenced a sharp decline in both the magnitude of its loading, as well as in its explanatory power (Δλ = −.754, Δ*R*^2^ = −.523), suggesting that the lexical alteration for this item was ultimately detrimental to the Revised model.^6^ In aggregate, the lexical alterations resulted in a slight decline in item loading magnitude (*M_change_* = −.048, *SD_change_* = .173, *min* = −.754, 25th *percentile* = −.072, *Median* = −.020, 75th *percentile* = .048, *max* = .154) and in the proportion of variance explained by the items (*M_change_* = −.041, *SD_change_* = .152, *min* = −.523, 25th *percentile* = −.093, *Median* = −.027, 75th *percentile* = .052, *max* = .24). For the CAPP-LRS model, several item loadings may be considered inadequately low depending on one’s criteria of judging them as such (e.g., Guadagnoli & Velicer, 1988; Hair et al., 1998). These items were “Lacks Anxiety” (λ = .221), “Sense of Uniqueness” (λ = .116), and “Restless” (λ = .308). Of these three items, the “Lacks Anxiety” item loading increased to λ = .298 when changed to “Unworried,” the “Restless” item loading increased to λ = .477 in the CAPP-Basic model (item not changed in CAPP-Basic), and the “Sense of Uniqueness” item loading decreased to .088 in the CAPP-Basic model (unchanged).^7^ As such, with the exclusion of the “Independent” item, no item in the CAPP-Basic model was found to have a problematically low item loading that was not already the case for the CAPP-LRS model. Overall, in both models the item loadings ranged from .5 to .8.

**Table 3.** Change Between CAPP-LRS and CAPP-Basic Item Loadings (λ), *R*^2^, and Item Discrimination Parameters (*a*).

CAPP-LRS				CAPP-Basic				Change^a^		
Item	λ	*R* ^2^	*a*	Item	λ	*R* ^2^	*a*	Δλ	Δ*R*^2^	Δ*a*
Dominance				Dominance						
Garrulous	0.721***	0.519	1.937	**Longwinded**	0.587***	0.345	1.178	0.134	−0.174	−0.759
Domineering	0.691***	0.477	1.912	**Bossy**	0.511***	0.261	1.121	0.180	−0.216	−0.791
Disruptive	0.692***	0.479	1.496	Disruptive	0.699***	0.489	1.959	−0.007	0.010	0.463
Unique	0.116	0.014	0.396	Unique	0.088	0.008	0.294	0.028	−0.006	−0.102
Reckless	0.654***	0.428	1.456	**Irresponsible**	0.703***	0.494	1.581	−0.049	0.066	0.125
Self-aggrandizing	0.708***	0.501	1.894	**Snooty**	0.686***	0.471	1.792	0.022	−0.030	−0.102
Intolerant	0.753***	0.567	1.556	**Unfair**	0.737***	0.544	2.011	0.016	−0.023	0.455
Invulnerable	0.522***	0.272	1.279	**Unbeatable**	0.535***	0.286	1.401	−0.013	0.014	0.122
Lacks anxiety	0.221***	0.049	0.333	**Unworried**	0.298***	0.089	0.686	−0.077	0.040	0.353
Manipulative	0.767***	0.588	1.911	**Tricky**	0.724***	0.524	1.683	0.043	−0.064	−0.228
Entitlement	0.651***	0.424	1.590	**Owed**	0.687***	0.471	1.637	−0.036	0.047	0.047
Aggressive	0.706***	0.499	1.922	**Nasty**	0.860***	0.739	2.843	−0.154	0.240	0.921
Self-centered	0.717***	0.514	2.012	Self-centered	0.658***	0.433	1.650	0.059	−0.081	−0.362
Disinhibition				Disinhibition						
Antagonistic	0.800***	0.640	1.709	**Unfriendly**	0.763***	0.583	1.090	0.037	−0.057	−0.619
Inflexible	0.479***	0.229	0.783	**Stubborn**	0.428***	0.183	0.716	0.051	−0.046	−0.067
Restless	0.308***	0.095	0.580	Restless	0.477***	0.228	0.914	−0.169	0.133	0.334
Self-justifying	0.609***	0.371	1.350	**Excuses**	0.682***	0.465	1.524	−0.073	0.094	0.174
Lacks planning	0.613***	0.376	1.616	**Chance**	0.554***	0.307	1.128	0.059	−0.069	−0.488
Lacks stability^b^	0.599***	0.359	1.405	Lacks stability^b^	0.558***	0.312	1.439	0.041	−0.047	0.034
Lacks concentration	0.593***	0.351	1.619	**Lacks focus**	0.600***	0.360	1.550	−0.007	0.009	−0.069
Perserverance	0.666***	0.444	1.320	**Gives up**	0.477***	0.227	1.246	0.189	−0.217	−0.074
Unstable self	0.669***	0.448	1.909	**Unsteady self**	0.678***	0.460	1.865	−0.009	0.012	−0.044
Deficient Attachment				Deficient Attachment						
Lacks pleasure	0.570***	0.325	1.237	Lacks pleasure	0.642***	0.412	1.508	−0.072	0.087	0.271
Lacks depth	0.567***	0.321	1.471	Lacks depth	0.648***	0.419	1.704	−0.081	0.098	0.233
Detached	0.665***	0.442	1.801	**Cold**	0.713***	0.508	1.886	−0.048	0.066	0.085
Suspicious	0.572***	0.327	0.970	Suspicious	0.622***	0.387	1.366	−0.050	0.060	0.396
Insincere	0.773***	0.597	1.888	**Fake**	0.750***	0.563	2.234	0.023	−0.034	0.346
Unreliable	0.681***	0.464	1.604	Unreliable	0.718***	0.516	1.604	−0.037	0.052	0
Unempathetic	0.848***	0.720	3.895	**Unfeeling**	0.739***	0.546	2.104	0.109	−0.174	−1.791
Lacks remorse	0.663***	0.439	1.578	**Shameless**	0.525***	0.276	0.785	0.138	−0.163	−0.793
Uncommited	0.724***	0.524	1.638	**Independent**	–0.030	0.001	−0.073	0.754	−0.523	−1.711
Uncaring	0.768***	0.590	2.932	**Unkind**	0.833***	0.694	2.650	−0.065	0.104	−0.282
Deceitful	0.767***	0.589	1.897	**False**	0.836***	0.699	2.861	−0.069	0.110	0.964

*Note.* Lexical alterations for the CAPP-Basic scale are bolded. Item loading and values were obtained from the three-factor confirmatory factor analytic models. Discrimination parameters were obtained from the domain-specific unidimensional graded-response models. CAPP-LRS = Comprehensive Assessment of Psychopathic Personality–Lexical Rating Scale.

a Where change is defined as CAPP-LRS estimates subtracted from CAPP-Basic estimates. ^b^Lacks *emotional* stability.

***p < .001. (Fisher, 1992).

#### Item Response Models

The domain-specific unidimensional graded-response models for the *Dominance* and *Deficient Attachment* factors exhibited strong fit to the data across all fit indices, however, the models for *Disinhibition* were found to have larger than ideal estimates of TLI and RMSEA. Nonetheless, we have included IRT comparisons for the *Disinhibition* items here, although, such comparisons should be interpreted with caution. Model fit statistics and indices for these models are presented in Table 4.

**Table 4.** CAPP-LRS and CAPP-Basic Model Fit for Unidimensional Graded-Response Models.

Model	*M_2_*	*df*	CFI	TLI	SRMR	RMSEA	RMSEA [90% CI]
Dominance							
CAPP-LRS	62.509***	39	0.972	0.963	0.059	0.042	[.021, .060]
CAPP-Basic	79.915***	39	0.942	0.922	0.065	0.055	[.038, .072]
Disinhibition							
CAPP-LRS	33.994***	10	0.938	0.883	0.079	0.083	[.053, .114]
CAPP-Basic	44.181***	9	0.910	0.820	0.072	0.103	[.076, .137]
Deficient Attachment							
CAPP-LRS	51.275***	24	0.972	0.959	0.071	0.057	[.035, .078]
CAPP-Basic	54.230***	22	0.955	0.955	0.066	0.065	[.043, .087]

*Note.* The *M_2_* statistic is limited to assessing dichotmous model fit, however, the *M_2_* statistic presented here is a variant of *M_2_* that accomodates polytomous data (see Cai & Hanson, 2013). CAPP-LRS = Comprehensive Assessment of Psychopathic Personality–Lexical Rating Scale; *df* = degrees of freedom; CFI = comparative index; TLI = Tucker–Lewis index; SRMR = standardized root mean residual; RMSEA = root mean square error of approximation; CI = confidence interval.

*** *p* < .001. (Fisher, 1992).

Similar to the CFA item loadings, visual inspection of the ICCs suggested a similar pattern of item responding between the CAPP-LRS and CAPP-Basic, for each of the three unidimensional factors. When changes in item responding were observed for the lexically altered items in the CAPP-Basic, the curves specific to each response category (i.e., category characteristic curves) tended to widen compared with their corresponding CAPP-LRS items, suggesting that those CAPP-Basic items were less discriminating. However, some CAPP-Basic items were noticeably more discriminating than their corresponding CAPP-LRS items (e.g., “Nasty,” “False”). The overall difference in discrimination between the CAPP-LRS and CAPP-Basic was, however, minimal in aggregate (*M_change_* = −.090, *SD*_change_ = .591, *min* = −1.791, 25th percentile = −.282, *Median* = 0, 75th *percentile* = .271, *max* = .964). When examining only the lexically altered items, more discrimination was lost than for the scales overall (*M_change_* = −.176, *SD_change_* = .655, *min* = −1.791, 25th percentile = −.521, *Median* = −.068, 75th percentile = .137, *max* = .964), however, the magnitude of the change was primarily driven by only two items, namely the Uncommitted/Independence *(*Δ*a* = −1.711) and the Unempathetic/Unfeeling *(*Δ*a* = −1.791) items. Although the lexical alteration from “Uncommitted” to “Independence” rendered the item unusable and problematic as noted elsewhere, the alteration from “Unempathetic” to “Unfeeling” still resulted in a useable and discriminating item. Item discrimination estimates are presented in Table 2.

**Figure 1.**
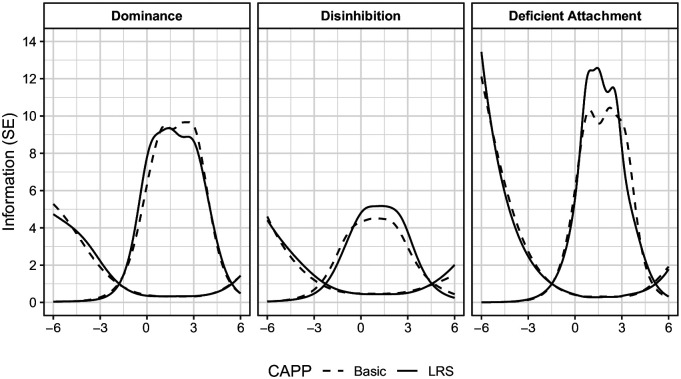
Test information curves and conditional standard error curves for the CAPP-LRS and CAPP-Basic Domains. *Note.* These curves were generated using parameter estimates from domain-specific unidimensional polytomous graded-response models for each CAPP domain. The conditional standard errors are plotted on the same scale as the test information curves.

Inspection of the IICs revealed generally similar distributions of information among the items. As would be expected, CAPP-Basic items that were less discriminating than their paired CAPP-LRS items (e.g., “Longwinded,” “Bossy,” “Chance,” “Unfriendly,” “Unfeeling,” and “Shameless”) also saw a loss of some information; albeit, their IICs spanned a larger range of theta than their corresponding CAPP-LRS items.^8^ At the level of the subscales, TICs and CSEMs were near-identical between the CAPP-LRS and CAPP-Basic models, as is shown in Figure 1.

### External Validity

Having found the internal psychometric properties of the CAPP-LRS and CAPP-Basic commensurate, we then compared estimates of criterion validity between the two via evaluation of patterns of association and response prediction on tools assessing traits commonly viewed as being within the nomological network of psychopathy (Miller & Lynam, 2012; Vachon et al., 2012). Both instruments exhibited generally moderate to strong bivariate relationships with measures of various types of aggression (e.g. physical, verbal, hostility; CAPP-LRS *rs =* .31 to .58; CAPP-Basic *rs* = .33 to .56), and impulsivity scores (CAPP-LRS *rs =* .29 to .46; CAPP-Basic *rs* = .25 to .46). Interestingly, for both instruments only the *Dominance* domain evidenced noteworthy associations with sensation seeking, with the *Deficient Attachment* domain exhibiting a nonsignificant association with this criterion for both instruments. From a general perspective, findings were overwhelmingly similar for both tools, with no meaningful difference in average correlation strength (*M*_difference_ = .02). Trends witnessed at the bivariate level were born out in a comparison of the predictive power of both instruments for participant self-report scores of outcome criteria. Specifically, results of OLS regressions controlling for the effects of gender and ethnicity suggested that each of the three CAPP domains was positively predictive of self-reported total aggression scores (CAPP-LRS: βs > .51, *ps* < .001; CAPP-Basic: βs > .48, *ps* < .001), and impulsivity (CAPP-LRS: βs > .20, *ps* < .001; CAPP-Basic: βs > .24, *ps* < .001). However, though all domains on the CAPP-Basic predicted sensation seeking (βs > .17, *ps* < .001), only the *Dominance* and *Disinhibition* domains were predictive of this trait for the CAPP-LRS (see Table 5).

**Table 5.** Ordinary Least Squares Regression Findings Controlling for Gender and Ethnicity.

CAPP	Aggression (BPAQ-SF)			Sensation seeking (BSSS)			Impulsivity (BIS-Brief)		
	B (*SE*)	β	Test Stat	B (*SE*)	β	Test Stat	B (*SE*)	β	Test Stat
*CAPP-LRS*									
Dominance	.84 (.08)	.52	*t*(3) = 11.28, *p* < .001	.35 (.05)	.36	*t*(3) = 6.70, *p*< .001	.19 (.03)	.34	*t*(3) = 6.65, *p* < .001
Deficient Attachment	.99 (.09)	.51	*t*(3) = 10.89, *p* < .001	.10 (.07)	.08	*t*(3)=1.51, *p* < .131	.19 (.04)	.28	*t*(3)=5.38, *p* < .001
Disinhibition	1.20 (.09)	.58	*t*(3) = 13.23, *p* < .001	.27 (.07)	.20	*t*(3) = 3.79, *p* < .001	.27 (.07)	.20	*t*(3) = 3.79, *p* < .001
*CAPP-Basic*									
Dominance	.84 (.08)	.48	*t*(3) = 10.23, *p* < .001	.29 (.06)	.26	*t*(3) = 5.00, *p* < .001	.21 (.03)	.35	*t*(3) = 6.81, *p* < .001
Deficient Attachment	.96 (.10)	.48	*t*(3) = 10.02, *p* < .001	1.00 (1.00)	.48	*t*(3)=10.02, *p*< .001	.17 (.04)	.24	*t*(3)=5.00, *p* < .001
Disinhibition	1.09 (.09)	.55	*t*(3) = 12.47, *p* < .001	.22 (.07)	.17	*t*(3) = 3.28, *p* < .001	.32 (.03)	.46	*t*(3) = 9.64, *p* < .001

### Descriptive Statistics and *t* tests

The final step in our analysis examined descriptive statistics for CAPP-LRS and CAPP-Basic total and subscale scores. Results indicated that observed score ranges were broadly similar for the CAPP-LRS and CAPP-Basic scales at the domain level (six-factor *M*_Diff_ = 1.7; three-factor *M*_Diff_ = 6), as were observed variances (six-factor *M*_Diff_ = 0.23; three-factor *M*_Diff_ = 2.95). Though maximum scores were highly similar for the majority of domains across the CAPP-LRS and CAPP-Basic scales, slightly lower minimum scores observed for the majority of domains on the CAPP-LRS. Such a finding suggests that, on average, individuals were more likely to rate themselves as possessing some level of the trait described when completing the CAPP-Basic scale, than when completing the CAPP-LRS (see Tables S2 and S3, available in the online supplement material).

In addition to descriptive comparisons, we also compared mean scores across both scales. Results of paired sample *t* tests suggested significant differences between scales for the *Dominance*, *Attachment* and *Cognitive* domains in the six-factor model (*t*s > |3.03|, *p*s < .01), and the *Deficient Attachment* and *Disinhibition* domains for the three-factor model (*t*s > 7.42, *p*s < .001). However, inspection of Cohen’s *d* values revealed that, with the exception of the *Attachment* domain (*d* = 1.2), the magnitude of differences between domains was generally small for both models (six-factor: *d*s < .22; three-factor: *d*s < .39).

## General Discussion

Results of a multilevel analytic strategy comparing the CAPP-LRS and CAPP-Basic from a descriptive, structural, and functional perspective at the item, domain, and test level revealed that the measures are defensibly interchangeable for practical use in research settings. This finding is important because it considerably expands the utility of the CAPP-LRS and broadens its scope in reaching samples of interest. Working from the fundamental to the more advanced level of comparison illuminates the overarching congruence between the CAPP-LRS and CAPP-Basic, but also requires the mention of noteworthy differences. For example, from a descriptive standpoint, the CAPP-Basic generally evidenced higher means than did the CAPP-LRS to a level that reached significance for two domains. In practical terms, this suggests that individuals were more likely to endorse trait descriptive symptoms on the CAPP-Basic than on the CAPP-LRS. Though differences in magnitude were generally small, this finding may be explained in part by a decrease in social desirability concerns on the CAPP-Basic, which uses more colloquial and common-place language instead of more nuanced descriptors. Though revised items may be less polarizing, this finding also represents a strength of the CAPP-Basic in that the social desirability concerns inherent in self-report, and especially salient when measuring socially aversive traits among more vulnerable populations (e.g., forensic, adolescent), are lessened with the use of this instrument. As such, this feature of the CAPP-Basic may actually represent one of its more desirable features, especially when evaluated alongside evidence that the variances and ranges of the CAPP-LRS and CAPP-Basic scales are markedly similar.

Comparison of the psychometric properties of the CAPP-LRS and CAPP-Basic was conducted via examination of the internal structure, domain, and item level functioning of each instrument. Congruent with previous findings in the literature, the originally proposed six-factor structure of the CAPP did not produce adequate fit to the data for either instrument in the present sample. However, in line with a growing body of research demonstrating the superiority of a three-factor solution to the CAPP framework (e.g., Flórez et al., 2018; Hanniball et al., 2020; Lim, 2016; Sellbom et al., 2015), we were able to confirm a well-fitting model reflecting first order facets of behavioral disinhibition, deficiency in emotional affect, and interpersonal dominance. Globally, this model provided relatively good fit to the data for the CAPP-LRS as well as the CAPP-Basic. When examining results of CFAs for these models across both instruments (e.g., *Dominance*, *Disinhibition*, and *Deficient Attachment*) very little discrepancy was observed in terms of model fit. Specifically, all models provided adequate to good fit to the data with the exception of the CAPP-LRS *Disinhibition* model, which evidenced suboptimal fit, but met standards for the CAPP-Basic. Closer examination of the item loadings for each domain on the CAPP-LRS and the CAPP-Basic similarly supports the functional similarity of the two instruments, suggesting a near-identical pattern of loading significance and similar pattern of loading magnitude for the vast majority of items, most of which exhibited moderate to strong loadings on each of the three domains (e.g. .5 to .8).^9^

We then contextualized these findings within an IRT framework to gain deeper understanding of item functioning across the latent continuum, evaluate item difficulty and discrimination and examine information provided at the test level. Results of IRT analyses paralleled those of the CFA investigation, again providing further justification for the use of the CAPP-Basic as a simplified parallel form of the CAPP-LRS. Beginning with evaluation of ICCs, which plot the probability of positive item response option endorsement across the continuum of the latent attribute, (e.g., *Dominance*), the pattern of item functioning is largely similar between the original CAPP-LRS and the CAPP-Basic. Specifically, items that function very well generally do so across both versions of the instrument, and relatively less discriminating items are also similar across both tools. Importantly, the majority of items on both instruments emerged from analyses as “good items” in the sense that they provided adequate discrimination (e.g., separated those with lower levels of the trait from those with higher levels of the trait), and those items that did not, for example “Sense of Uniqueness” and “Lacks Anxiety” are items that have proved problematic for the CAPP model in past research as well (Hanniball, et al., 2020). In cases where we did witness some difference between the two scales, the magnitude of change was negligible. For example, though some items resulted in some loss of discrimination, or wider curves (e.g., “Garrulous” to “Longwinded”), certain items were more discriminating, or information was more targeted (e.g., “Aggressive” to “Nasty”).

ICCs, which indicate how precisely each item measures the trait at various levels of the attribute, were also markedly similar across the CPP-LRS and CAPP-Basic. Broadly speaking, items on both instruments generally provided more information at the higher, or above average ranges (e.g., more information provided for those “high” on psychopathy), than at the lower ranges. Though this is not necessarily a weakness of the CAPP instruments given that the population of interest when implementing these tools is often comprises of individuals who fall at the higher ends of the latent continuum, it also suggests that when such tools are utilized in research settings where the range and distribution of psychopathic traits is restricted to the lower ends (e.g., university students, community samples) they may provide less information than desirable. This introduces the possibility of adding in further items that aim to obtain more information at the lower or “below average” range, especially if a large portion of the research conducted with such tools is among the aforementioned populations, as has been largely true of previous CAPP research.

Perhaps most important to this context, attention to TICs, which reveal how well the test as a whole measures the latent trait across various levels of the attribute, suggests that at the test level, the CAPP-LRS and CAPP-Basic scales are virtually indistinguishable and reflect the same anchor points, even when broken down into the three domains that constitute the scale. At the aggregate level, both the CAPP-LRS and CAPP-Basic provide the most information and precision in measurement for individuals who fall at the higher ends of the psychopathy continuum; that is, those who are more interpersonally dominant and aggressive, those who are more behaviorally disinhibited, and those who possess lower levels of emotional affect. Given that the aim of developing the revised scale is to make the CAPP-LRS more suitable for use among populations that may arguably contain higher rates of psychopathic traits and concurrently possess lower levels of verbal ability (e.g., forensic samples), this finding represents a strength in the current context.

As expected, given the results of psychometric analyses, lexical alteration to the CAPP-LRS did not alter its validity with respect to pattern of association and response prediction for well recognized traits associated with psychopathy. In particular, both instruments evidenced moderate to strong positive relationships with physical and verbal aggression, anger, hostility, and impulsivity. Furthermore, the relationship between each instrument and sensation seeking tendencies was also markedly similar, suggesting that symptoms associated with interpersonal dominance and aggression, as well as those associated with behavioral disinhibition are positively related to sensation seeking. However, for both instruments, the presence of deficient affect was found to be weakly related or unrelated to preference and pursuit of high arousal thrills. This finding is interesting not only in its reflection of the functional similarity of the CAPP-LRS and CAPP-Basic but also provides evidence for specificity of symptom clusters reflective of psychopathic personality pathology. Theoretically speaking, this result makes sense, and fits with trends in the literature which suggest that aggression, interpersonal dominance, impulsivity, and decreased urge inhibition are associated with experience and thrill seeking (Hur & Bouchard, 1997; Trimpop et al., 1999; Wilson & Scarpa, 2011). On the other hand, from a theoretical perspective, deficits in emotional processing, or decreased experience of emotional valence may be less strongly associated with the desire for activities that provide high arousal rewards given evidenced differences in response patterns associated with emotional stimuli (Loney et al., 2003). As such, the pattern of association between relevant outcome criteria and both versions of the CAPP is consistent with expectations and suggests support for congruent construct-related validity.

Despite overwhelming similarities with respect to test and item functioning, one significant and notable exception is worthy of note. In line with findings from the pilot study and initial item selection screen, the original CAPP symptom “Uncommitted,” which loads onto the *Deficient Attachment* factor, was changed to “Independent” in the CAPP-Basic. Across all comparative evaluations investigated under both a CFA and IRT framework, this item emerged as significantly different in terms of its functioning, fit, and utility as compared to the original wording. Indeed, this item performed so poorly, that its inclusion in the CAPP-Basic resulted in a significant depreciation in the fit of the overall CFA model, and drove the majority of the average change in item loading strength across the two versions of the scale. IRT analyses further confirmed these findings, suggesting inferior and unacceptable item performance with respect to discrimination and measurement precision. As such, this item should be omitted or replaced with the original CAPP-LRS item when implementing the CAPP-Basic in research settings, with findings suggesting that the original wording (“Uncommitted”) is preferable for practical use.

## Limitations and Future Directions

Though this investigation provides promising initial validation results for the lexically simplified parallel version of the CAPP-LRS, this research is not without limitations. First, though this work represents only the first step in the development and validation of the CAPP-Basic, conclusions drawn in the present research are constrained by the project’s reliance on a convenience sample (e.g., university students). As such, this research can only be said to represent a preliminary step rather than the final word on the validation of the CAPP-Basic. The goal of the pilot study and initial adaptation process was to produce a lexically sheltered version of the CAPP-LRS to make it more suitable for use among individuals possessing lower verbal and reading skills. Given that university students likely possess average to above average language skills, it is possible that perceptions of what constitutes simplified language were skewed in this study. This being said, the multistep item selection process, statistically guided decision-making procedure, and consultation with linguistic experts help to alleviate these concerns. Furthermore, establishing the performance equivalence of the simplified (Capp-Basic) and more complex (CAPP-LRS) instruments among the populations that with higher language skills represents a crucial primary step to establish the validity of the adapted tool, allowing us to address the question of functional equivalence directly.

Confidence is further bolstered by the results of formal tests of grade level readability for the CAPP-LRS as compared with the CAPP Basic. Specifically, while the CAPP-LRS requires an estimated 20.4 years of education for reasonable comprehension of its items, the CAPP-Basic requires only 8.6 years of education as estimated by the Flesch–Kincaid Grade Level Test, representing a reduction of educational requirements of roughly 12 years, or 57.84%. The Flesch–Kincaid Tests are well recognized adjudicators of text difficulty and are relied on in both military and legal settings. These tests have been shown to be reliable, valid, and exhibit high levels with other established readability scales (Kincaid et al., 1975; Li & Callan, 2001; McClure, 1987). This being said, we must emphasize the necessity for further research to validate the CAPP-Basic within the populations that inspired its design. Critically, until such research has been conducted, we cannot recommend the CAPP-Basic for applied use within these populations.

Second, though results of this initial investigation provide good evidence for the psychometric properties of the CAPP-Basic and its structural and functional similarity to the CAPP-LRS, prototypicality studies with mental health experts that directly evaluate the content validity of the symptom translations in the lexically modified instrument are needed to further confirm its value. Fortunately, such an investigation is already underway. Recent prototypicality work by Hanniball et al. (in press), suggests that mental health experts and forensic specialists view the majority of revised items included in the CAPP-Basic to be highly prototypical of psychopathy. In addition, findings regarding the specificity of items was shown to closely parallel that of previous prototypicality work with the CAPP-LRS.

Third, though we were able to address the question of functional equivalence in the present work because all participants completed both versions of the instrument, this is unfeasible when assessing samples of specific interest who can only engage with the simplified version of the tool (e.g., youth, forensic samples). This issue creates somewhat of an asymmetry in establishing the parallel nature of the CAPP-Basic. However, future research can address these questions, albeit in an indirect manner, in evaluating the pattern of evidence, correlations, and associations of the CAPP-Basic alongside external criteria of theoretical relevance to psychopathy, as well as in conducting comparative examinations between the CAPP-Basic and other established measures of psychopathy within these samples (e.g., CAPP-IRS; PCL-R; PCL-YV). In addition, though it was beyond the scope of the current investigation to evaluate the invariance of the present findings across important demographic characteristics (e.g., gender, ethnicity), an imperative step for future research with the CAPP-Basic will be to evaluate its psychometric properties, validity, and utility for use across diverse samples.

A fourth limitation pertains to the generalizability of structural and psychometric findings given the nature of the current sample (e.g., university undergraduates). However, the use of IRT methodology, alongside classical test theory analyses (e.g., CFA) mitigates this concern. Specifically, though CFA methodology is largely dependent on the items included in the test and the persons examined, meaning that conclusions are sample reliant, specifics of IRT methodology allow more confidence in the generalizability of findings. For example, IRT overcomes the item-person confound problem inherent in CFA analyses by yielding estimates of item difficulties and person abilities that are independent of each other (Bond & Fox, 2001). Furthermore, when using CFA, the standard error of measurement is averaged across persons in the sample and is thereby specific to that population or sample. IRT overcomes this obstacle because under this methodology, the standard error of measurement is considered to vary across scores in the same population, and to be population general (Embretson & Reise, 2000). As a result, under IRT, precision in measurement can be evaluated at any level of the latent trait, instead of averaged over trait levels as when using classical test theory methods (Hambleton et al., 1991). As such, though further work is clearly needed to replicate the findings of the present research, the data analytic strategy employed here allows for increased confidence in the robust nature of our findings given the sample independent nature of IRT.

The final limitation of the present work concerns the online sampling method, offering less control than protocols administered in-person, and introducing greater possibility of distraction and inattentive responding. However, though online protocols have some drawbacks, they also provide some strengths, particularly when measuring socially undesirable personality features such as those associated with psychopathy. Specifically, though the majority of traits assessed in the present study are vulnerable to response bias and dishonesty (Sellbom et al., 2018), research has demonstrated that in an anonymous research setting (such as is inherent in online sampling protocols) response bias is less likely to affect the validity of self-reports (Watts et al., 2016). Finally, though we would have liked to include a wider range of criterion variables in our study, we were limited in balancing brevity and onus on participant time. Future work should aim to assess correlations of the CAPP-Basic with other measures specific to the construct of psychopathy and personality, such as indices of the five-factor model or HEXACO model (e.g., NEO-PI-R; HEXACO-PI-R).

Notwithstanding these limitations, results of the present investigation indicate that though linguistic demands are lowered in the CAPP-Basic, it remains functionally equivalent to the original scale in terms of its psychometric properties and test function. Though future research is needed to more fully validate the CAPP-Basic, this project represents a promising first step, and adds to the greater body of literature supporting the robust nature of the CAPP framework.

## Supplemental Material

sj-pdf-1-asm-10.1177_10731911211040108 – Supplemental material for Increasing the Utility of the Comprehensive Assessment of Psychopathic Personality–Lexical Rating Scale (CAPP-LRS): Instrument Adaptation and Simplification Click here for additional data file. Supplemental material, sj-pdf-1-asm-10.1177_10731911211040108 for Increasing the Utility of the Comprehensive Assessment of Psychopathic Personality–Lexical Rating Scale (CAPP-LRS): Instrument Adaptation and Simplification by Katherine B. Hanniball, Richard Hohn, Erin K. Fuller and Kevin S. Douglas in Assessment

## Notes

1 It is noted that as of the date of this publication, a prototypicality study conducted with the CAPP-Basic is already in press at *Journal of Personality Disorders.* Findings of this investigation suggest support for the content validity of the CAPP-Basic, and indicate that results regarding symptom and domain prototypicality ratings, as well as symptom specificity ratings, largely parallel results observed in past studies (Hanniball et al., in press).

2 Considerable debate exists as to whether the psychopathy construct can be suitably applied to children and adolescents. Relevant concerns include the stability of psychopathy over time, the predictive validity of youth psychopathy assessment for maladaptive outcomes in adulthood, the malleability of trait expression during the child and adolescent developmental period, and the stigmatizing effects of the psychopathy label (Edens & Vincent, 2008; Seagrave & Grisso, 2002; Shepherd & Strand, 2016). Despite these concerns, general consensus in the field supports the importance of research assessing trait development across the lifespan and identifying stable correlates of psychopathic personality disorder in children and adolescents (Corrado et al., 2004; Frick et al., 2014; Ridder & Kosson, 2018).

3 Estimate drawn from results of the of the Flesch–Kincaid Grade Level Test, which is a readability test designed to indicate the grade level required for understanding of written text. The formula on which the Flesch–Kincaid relies (i.e., the F-K formula) is well recognized and is a requirement in numerous states for many legal documents in order to ensure widespread understanding (McClure, 1987).

4 Evidence of a refined three-factor CAPP model has been articulated in the recent work cited here.

5 Some previous research has indicated that the pattern of external correlates of psychopathy may differ on account of gender and ethnicity, suggesting the importance of controlling for these variables in the present analyses (e.g., Jackson et al., 2007; Knight et al., 2002; Sullivan et al., 2006).

6 In fact, for the CAPP-LRS, this item was responsible for a sizeable portion of the decline in model fit. When the “Independent” item is removed from the analysis, the model fit improves to χ^2^ = 1105.334, *df* = 491, *p <* .001, CFI = .919, TLI =.913, SRMR = .081, RMSEA = .063, RMSEA 90% CI [.059, .068].

7 Further note that Hanniball et al. (2020) observed the “Sense of Uniqueness” item to be problematic in their evaluation of several CAPP-LRS model factor structures and ultimately decided to remove it from their analyses.

8 Readers interested in graphical representations of ICCs and IICs as well as parameter estimates are encouraged to visit the project as posted on the OSF using the following link: https://osf.io/rwueb/

9 Exceptions included the items “Sense of Uniqueness,” “Lacks Anxiety,” and “Restless.” Notably, linguistic revisions generally increased strength of these items; however, previous research into the psychometric properties of the CAPP has also indicated problems with these items (Hanniball et al., 2020).
