# Supplementary material for: Increasing the Utility of the Comprehensive Assessment of Psychopathic Personality–Lexical Rating Scale (CAPP-LRS): Instrument Adaptation and Simplification
Source: Assessment. 2021 Aug 20;29(8):1954–72. doi: 10.1177/10731911211040108 (PMC9596956; doi:10.1177/10731911211040108)
Supplement: sj-pdf-1-asm-10.1177_10731911211040108 – Supplemental material for Increasing the Utility of the Comprehensive Assessment of Psychopathic Personality–Lexical Rating Scale (CAPP-LRS): Instrument Adaptation and Simplification [file sj-pdf-1-asm-10.1177_10731911211040108.pdf]

Supplemental Material

Table S1

Initial Difficulty and Similarity Screen

Original Item	Domain	Replacement Items ($M_{readability}$; $M_{similarity}$)
Self-Aggrandizing	Self	Snooty (3.86; 4.02), Big-headed (3.00; 3.24)
Sense of Entitlement	Self	Sense of being owed things (2.39; 3.08), Sense of worthiness (2.94; 2.67)
Sense of Invulnerability	Self	Sense of being unbeatable (2.71; 3.47), Sense of being indestructible (4.22; 3.14)
Self-Justifying	Self	Offers a lot of excuses for behavior (2.61; 4.10), Offers a lot of reasons for behavior (2.82; 3.98)
Unstable Self-Concept	Self	Unsteady sense of self (3.24; 3.08), Uncertain sense of self (2.65; 2.33)
Lacks Anxiety	Emotional	Unworried (2.43; 2.08)
Lacks Remorse	Emotional	Shameless (2.80; 3.33), Lacks regret (2.65; 2.73)
Antagonistic	Dominance	Unfriendly (1.47; 3.65), Hostile (3.55; 2.92)
Domineering	Dominance	Overbearing (3.80; 3.31), Bossy (1.45; 3.27)
Deceitful	Dominance	False (2.24; 4.16), Two-faced (3.59; 3.16)
Manipulative	Dominance	Tricky (2.04; 3.45)
Insincere	Dominance	Fake (1.67; 2.49)
Garrulous	Dominance	Long-winded (2.88; 3.96), Wordy (2.73; 3.06)
Detached	Attachment	Cold (2.00; 3.63), Removed (2.94; 2.53)
Uncommitted	Attachment	Independent (2.55; 3.94), Uninterested (2.88; 2.63)
Unempathetic	Attachment	Unrelatable (3.31; 3.29), Unfeeling (2.88; 3.29)
Uncaring	Attachment	Cold (2.33; 2.69), Unkind (2.73; 2.51)
Lacks Perseverance	Behavioral	Lacks purpose (3.02; 3.57), Gives up easily (1.45; 3.06)
Reckless	Behavioral	Thoughtless (3.14; 3.08), Irresponsible (2.96; 3.00)
Aggressive	Behavioral	Nasty (2.27; 3.86)
Lacks Concentration	Cognitive	Lacks focus (2.71; 2.20)
Intolerant	Cognitive	Unfair (1.98; 3.29), Biased (3.12;2.82)
Lacks Planfulness	Cognitive	Stubborn (1.86; 2.43)

Note. Higher means indicate harder difficulty rankings (range: 1-5); higher means indicate more similar items (range: 1-5).

Table S2

Descriptive Analysis Six-Factor Model

	Six-Factor CAPP-LRS					Six-Factor CAPP-Basic				
	Minimum	Maximum	Mean	*SD*	Variance	Minimum	Maximum	Mean	*SD*	Variance
Attachment	4.00	12.00	5.56	1.93	3.72	3.00	13.00	7.48	1.89	3.57
Behavioral	3.00	19.00	9.73	2.76	7.50	6.00	20.00	9.62	2.74	7.52
Cognitive	3.00	18.00	9.28	2.76	7.53	5.00	17.00	9.87	2.65	7.02
Dominance	6.00	22.00	9.11	3.07	9.42	6.00	18.00	9.47	2.82	9.61
Emotional	4.00	15.00	8.43	2.36	5.51	5.00	16.00	8.40	2.38	5.66
Self	7.00	23.00	12.48	3.35	11.24	7.00	24.00	12.82	3.30	10.91

Note. SD = standard deviation.

Table S3

Descriptive Analysis Three-Factor Model

	Three-Factor CAPP-LRS					Three-Factor CAPP-Basic				
	Minimum	Maximum	Mean	*SD*	Variance	Minimum	Maximum	Mean	*SD*	Variance
Dominance	6.00	39.00	21.10	5.79	33.51	13.00	39.00	21.30	5.36	28.77
Deficient Attachment	2.00	32.00	16.28	4.81	23.16	11.00	33.00	18.15	4.61	21.28
Disinhibition	3.00	18.00	9.28	2.76	7.53	5.00	17.00	9.87	2.65	7.02

Note. SD = standard deviation.
